# A randomized, phase III trial of capecitabine plus bevacizumab (Cape-Bev) versus capecitabine plus irinotecan plus bevacizumab (CAPIRI-Bev) in first-line treatment of metastatic colorectal cancer: The AIO KRK 0110 Trial/ML22011 Trial

**DOI:** 10.1186/1471-2407-11-367

**Published:** 2011-08-23

**Authors:** Clemens Giessen, Ludwig Fischer von Weikersthal, Axel Hinke, Sebastian Stintzing, Frank Kullmann, Ursula Vehling-Kaiser, Julia Mayerle, Markus Bangerter, Claudio Denzlinger, Markus Sieber, Christian Teschendorf, Jens Freiberg-Richter, Christoph Schulz, Dominik Paul Modest, Nicolas Moosmann, Philipp Aubele, Volker Heinemann

**Affiliations:** 1 Department of Medical Oncology, Klinikum Grosshadern, University of Munich, Germany; 2 MVZ Gesundheitszentrum St. Marien, Amberg, Germany; 3 WISP GmbH, Langenfeld, Germany; 4 Department of Internal Medicine I, Klinikum Weiden, Germany; 5 Oncological Practice, Landshut, Germany; 6 Department of Medicine, University Hospital Greifswald, Germany; 7 Oncological Practice Augsburg, Germany; 8 Department of Medicine III, Marienhospital, Stuttgart, Germany; 9 Department of Medicine II, Gummersbach Hospital, Gummersbach, Germany; 10 St.-Josefs-Hospital Dormund-Hörde, Germany; 11 Oncological Practice, Dresden, Germany

## Abstract

**Background:**

Several randomized trials have indicated that combination chemotherapy applied in metastatic colorectal cancer (mCRC) does not significantly improve overall survival when compared to the sequential use of cytotoxic agents (CAIRO, MRC Focus, FFCD 2000-05). The present study investigates the question whether this statement holds true also for bevacizumab-based first-line treatment including escalation- and de-escalation strategies.

**Methods/Design:**

The AIO KRK 0110/ML22011 trial is a two-arm, multicenter, open-label randomized phase III trial comparing the efficacy and safety of capecitabine plus bevacizumab (Cape-Bev) versus capecitabine plus irinotecan plus bevacizumab (CAPIRI-Bev) in the first-line treatment of metastatic colorectal cancer. Patients with unresectable metastatic colorectal cancer, Eastern Cooperative Oncology Group (ECOG) performance status 0-1, will be assigned in a 1:1 ratio to receive either capecitabine 1250 mg/m^2 ^bid for 14d (d1-14) plus bevacizumab 7.5 mg/kg (d1) q3w (Arm A) or capecitabine 800 mg/m^2 ^BID for 14d (d1-14), irinotecan 200 mg/m^2 ^(d1) and bevacizumab 7.5 mg/kg (d1) q3w (Arm B). Patients included into this trial are required to consent to the analysis of tumour tissue and blood for translational investigations. In Arm A, treatment escalation from Cape-Bev to CAPIRI-Bev is recommended in case of progressive disease (PD). In Arm B, de-escalation from CAPIRI-Bev to Cape-Bev is possible after 6 months of treatment or in case of irinotecan-associated toxicity. Re-escalation to CAPIRI-Bev after PD is possible. The primary endpoint is time to failure of strategy (TFS). Secondary endpoints are overall response rate (ORR), overall survival, progression-free survival, safety and quality of life.

**Conclusion:**

The AIO KRK 0110 trial is designed for patients with disseminated, but asymptomatic mCRC who are not potential candidates for surgical resection of metastasis. Two bevacizumab-based strategies are compared: one starting as single-agent chemotherapy (Cape-Bev) allowing escalation to CAPIRI-Bev and another starting with combination chemotherapy (CAPIRI-Bev) and allowing de-escalation to Cape-Bev and subsequent re-escalation if necessary.

**Trial Registration:**

ClinicalTrials.gov Identifier NCT01249638

EudraCT-No.: 2009-013099-38

## Background

Colorectal cancer (CRC) is the second leading cancer entity in Germany with an incidence of approximately 71.000 and about 30.000 deaths every year. With a median age of about 70 years, many elderly patients are affected by this disease. In about 20% of patients synchronous metastasis is apparent at first diagnosis, while 20% to 25% of patients develop metachronous metastasis. Unfortunately, only 10% to 20% of mCRC patients are resectable at the time of presentation [1,2].

Three major groups of mCRC patients can be differentiated: 1. Patients with resectable colorectal cancer. 2. Patients with potentially resectable metastasis that require intensive combination therapy to convert the disease to a resectable state. Combination chemotherapy is also necessary in patients with symptomatic or rapidly progressive disease. 3. Patients with disseminated multiple metastases, who are not potential candidates for resection and who present with mostly asymptomatic, not rapidly progressing disease. These patients do not necessarily benefit from rapid remission induction or high overall response rates. So far, most randomised trials have not aimed to clearly separate these groups in order to apply distinctly different therapies.

Therefore, less intensive regimens focusing on survival and disease control may be a better choice for first-line treatment in these patients. Grothey et al. analyzed the AVF2107g and N9741 trial and identified tumour response not as a necessary factor to provide benefit to an individual patient in first-line therapy for metastatic colorectal cancer (mCRC). Although patients achieving response had a better prognosis, response was not predictive of the benefit derived from the superior treatment in either trial [3].

The combination of a fluoropyrimidine plus bevacizumab was previously shown to be effective in the first-line treatment for mCRC and demonstrated progression-free survival times of 8 to 9 months and disease control rates (DCR) of 69%-92.5% [4,5]. Also low rates of progressive disease (<10%) have been reported in this treatment regimen.

The use of the oral fluoropyrimidine capecitabine in combination with bevacizumab was previously shown to be safe and effective in the first-line treatment of mCRC. In a recent report, this combination allowed a DCR of 92% and a PFS of 8.5 months [5]. By comparison, the combination of capecitabine with irinotecan (CAPIRI) plus bevacizumab induced a disease control rate of 72%-82% and a PFS of 9-12 months [6,7]. Earlier trials evaluating chemotherapy regimen with capecitabine and irinotecan had reported unacceptable incidences of severe gastrointestinal adverse effects with grade 3/4 diarrhea up to 36% of patients [8-10]. We therefore decided for a 20% dose reduction (i.e., capecitabine 800 mg/m2, irinotecan 200 mg/m2) previously investigated in two AIO randomized phase II trials [7,11]. Acceptable gastrointestinal toxicity with grade 3-4 diarrhoea occurring in 15.7%-21.0% of patients has been reported for the combination of capecitabine and irinotecan [7,9,11]. The data from the two AIO trials also demonstrated that while both, CAPOX and CAPIRI, regimens are highly active and safe, the absence of sensory neuropathy favours CAPIRI as the chemotherapy backbone for bevacizumab in this first-line mCRC trial.

The AIO KRK 0110 trial investigates the combination of capecitabine and bevacizumab (Cape-Bev) versus the combination of capecitabine, irinotecan bevacizumab (CAPIRI-Bev). This trial is designed to investigate two different treatment strategies for unresectable, disseminated, but asymptomatic metastatic colorectal cancer patients with the goal of long-term disease stabilization and moderate toxicity.

In case of first occurrence of progressive disease (PFS-1) in the Cape-Bev-arm, treatment is escalated by adding irinotecan (CAPIRI-Bev). PFS-2 can be investigated in patients developing SD or PR/CR after treatment intensification.

In the comparator arm, patients receive CAPIRI + bevacizumab (CAPIRI-Bev) as first-line therapy. De-escalation to Cape-Bev is allowed after 6 months of treatment or in case of irinotecan-induced toxicity. The primary endpoint is the time to failure of strategy (TFS). Toxicity will be evaluated according to NCI CTC using a predefined score system accounting for symptomatic grade 2-4 toxicities per cycle in case of comparable TFS times.

Quality of life assessment is performed in both treatment arms to investigate the impact of monochemotherapy and combination chemotherapy during first-line therapy. Vice versa, differences in treatment efficacy and reduction of cancer-related symptoms will be correlated by quality of life analyses using the EORTC-QLQ-C30 questionnaire.

## Methods/Design

### Primary objective

**Figure 1:**
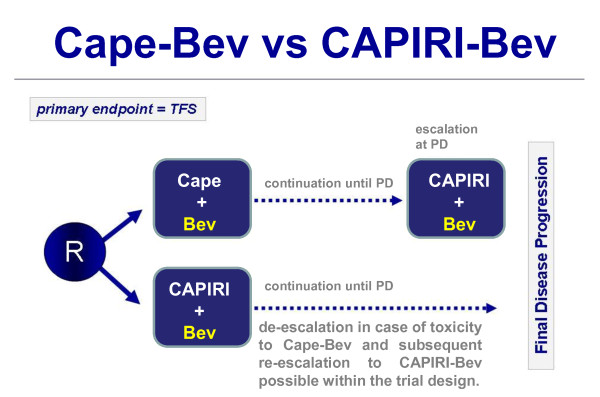
**Flow chart of the AIO KRK 0110 Trial/ML22011 Trial**. Patients are randomized to either receive single-agent chemotherapy (Cape-Bev) and escalation to CAPIRI-Bev at progressive disease or combination chemotherapy (CAPIRI-Bev) with an de-escalation option to Cape-Bev and subsequent re-escalation if necessary.

The primary objective is to investigate the efficacy of both treatment strategies in the first-line treatment of patients with unresectable mCRC. Since the sequential application of treatment regimens is evaluated, time to failure of strategy (TFS) was chosen as the primary endpoint (Figure 1).

In the Cape-Bev arm, treatment may be escalated after disease progression (PFS-1) by addition of irinotecan (CAPIRI-Bev); PFS-2 can then be investigated in patients after treatment intensification. In the experimental Cape-Bev arm, time to failure of strategy (TFS) is defined as PFS-1 + PFS-2, where PFS-1 is the time between randomization and first failure of Cape-Bev, and PFS-2 is the time between PFS-1 and PD after treatment intensification to CAPIRI-Bev.

In the CAPIRI-Bev comparator arm, treatment may be de-escalated to Cape-Bev and re-escalated to CAPIRI-Bev according to a predefined algorithm. TFS is defined as the time between randomization and progression on the CAPIRI-Bev treatment strategy.

In case of comparable TFS times in both study arms, toxicity will be evaluated according to NCI CTC in a predefined score system using symptomatic grade 2-4 toxicities per cycle.

### Secondary objective

Secondary objectives are to compare overall response rate (ORR), PFS-1, PFS-2 (experimental arm only), overall survival (OS), safety, toxicity and quality of life. Survival is defined as the interval between randomization and death of any cause. The grade of toxicity will be assessed using the NCI-CTC criteria version 4.0. Quality of life will be studied by means of the EORTC QLC C30 questionnaire.

### Design

The AIO KRK 0110 ML22011 trial is a two-arm, multicenter, open-label randomized phase III trial comparing the efficacy and safety of capecitabine plus bevacizumab versus capecitabine plus irinotecan plus bevacizumab in the first-line treatment of metastatic colorectal cancer. This trial is designed for patients with disseminated, but asymptomatic metastatic disease in good general condition (ECOG 0-1) who may not benefit from aggressive first-line treatment according to the definition of group-3 defined in the German S3-guidelines for colorectal cancer [12,13].

### Enrolment

A total of 516 patients will be enrolled in a 1:1 randomization into the two treatment arms. Patients will be enrolled in 80 to 100 study centres. The study started on December, 21^st ^2010. The estimated primary completion date is December 2013 (Final data collection date for primary outcome measure) and the estimated study completion date is December 2016.

### Stratification

Treatment assignment will be stratified, based on:

1. Prior adjuvant chemotherapy or no prior adjuvant chemotherapy

2. White-blood-cell count <8.000/μl vs. > 8.000/μl

3. Alkaline phosphatase <300 U/l vs. > 300 U/l

## Eligibility criteria

### Inclusion criteria

• Written informed consent

• Histologically confirmed adenocarcinoma of the colon or rectum

• Stage IV disease considered irresectable or patient declining surgery

• No prior chemotherapy for metastatic disease

• Metastatic disease evaluable according to RECIST version 1.1 (CT scan of thorax and abdomen within 4 weeks before randomization)

• Age 18 years and older

• ECOG 0-1

• Life expectancy > 3 months

• Assessment of KRAS status (local assessment, result not requested at time of randomization)

• Patient agreed storage of tumour tissue for molecular analyses and genetic profiling

• Time to previous adjuvant chemotherapy > 6 months

• No surgery within 28 days prior to treatment start. No cytological biopsies, implantation of venous port system within 7 days prior to treatment start. Operation sequels need to be completely healed. Major operations must not be expected at time of study begin, except for potential secondary resection of liver metastases. In case of secondary resection of liver metastases, bevacizumab must be discontinued 6-8 weeks prior to surgery.

• Effective contraception in childbearing potential women

• Precluded pregnancy

• Normal cardiac function (ECG and cardiac ultrasound) with LVEF >= 55%

• INR <1.5 and APTT <1.5 ULN in patients without anticoagulant medication. Therapeutic anticoagulation is allowed if INR and APTT are in the therapeutic range and stable for at least 2 weeks

• Continuous medication with acetylsalicylic acid is allowed up to a daily dose of 325 mg. Clopidogrel is allowed up to a recommended daily dose of 75 mg. Ticlodipine is allowed up to a recommended daily dose of 500 mg. Combination of acetylsalicylic acid and clopidogrel or ticlodipine is not allowed.

• Urine dipstick < 2+ for protein. In patients with >= 2 + protein a 24-hour-urine collection should be performed and proteinuria should be <= 1 g/24 h

• Adequate hematologic function: leukocytes >= 3000/μl, neutrophils >= 1500/μl, platelets >= 100.000/μ, and haemoglobin >= 9 g/dl.

• Adequate hepatic function: Bilirubin <= 1,5× upper limit of normal (ULN). ALAT and ASAT <= 2,5× ULN, in case of liver metastases <= 5× ULN

• Adequate renal function: Serum creatinine <= 1,5× ULN or creatinine clearance (GFR Cockroft and Gault >= 50 ml/min)

### Exclusion Criteria

• Primary resectable metastases or patients requesting surgical intervention

• Heart failure Grade III/IV (NYHA-classification)

• Medical history or pre-existing condition making the patient ineligible for study participation or interfering the patients safety

• Myocardial infarction, unstable angina pectoris, balloon angioplasty (PTCA) with or without stenting within the last 6 months

• Medical history of arterial thromboembolic events including apoplectic stroke, transient ischemic attack or cerebrovascular disorder within the last 6 months

• Severe bleeding event within the last 6 months (except tumour bleeding surgically treated by tumour resection), coagulopathy, haemorrhagic diathesis

• Abdominal or tracheo-esophageal fistulas, gastrointestinal perforation within 6 months before study entry

• Uncontrolled hypertension defined as systolic blood pressure >150 mm Hg and/or diastolic > 100 mm Hg under antihypertensive medication.

• Medical history of recurrent thromboembolic events (> 1 episode of deep vein thrombosis, peripheral embolism) within the last 2 years.

• Severe chronic wounds, ulcerous lesions or bone fracture.

• Pregnant or breast feeding women (pregnancy needs to be excluded by testing of beta-HCG).

• Medical, psychiatric, familial, sociological or geographical condition which contradicts participation of study

• Additional cancer treatment (chemotherapy, radiotherapy, phytotherapy, immunotherapy, or hormonal treatment) during study

• Current treatment with another investigational drug, any other prohibited drug or participation in another investigational study

• Contraindication for irinotecan treatment

• Known acute or delayed allergy or idiosyncrasy against capecitabine, irinotecan and bevacizumab and chemical related drugs

• Acute or subacute bowel obstruction, chronic inflammatory bowel disease or chronic diarrhea

• Known glucuronidation-deficiency (Gilbert-Meulengracht-Syndrome) (special screening not required)

• Non-treated cerebral metastases

• Medical history of other malignant disease within 5 years prior to study entry, except for basalioma, and in-situ cervical carcinoma if treated with curative intent

• Known alcohol or drug abuse

• Limited legal capacity

### Randomization

After verification of all eligibility criteria, stratification parameters and having obtained patient's written informed consent, patients will be randomized by fax at the monitoring office (ClinAssess GmbH fax +49-2171-3633655). Randomized treatment will be confirmed by fax within one (1) working day. Randomization takes place with balanced block design using PROC PLAN (SAS Institute Inc., Cary, NC USA).

### Ethics

This study is conducted according to the standards of Good Clinical Practice (GCP), and in agreement with the latest revision of the Declaration of Helsinki (2000, including the notes of clarification 2002, 2004 and 2008) and local regulations. This protocol has been submitted and approved by the Ethical Committee (EC) of the University of Munich - Faculty of Medicine http://www.ethikkommission.med.uni-muenchen.de/index.html.

The independent medical ethics committees of all participating hospitals have approved the study protocol. The EC may be notified of administrative changes and Suspected Unexpected Serious Adverse Reaction (SUSAR). Oral and written informed consent in form is obtained from all patients prior to randomization.

### Stopping rules

If excess harm is observed, or if a statistically significant benefit (resulting from a pre-specified sequential analysis procedure) is observed, the study is stopped and the patient is informed of the results. Also the trial can be stopped after consideration of justified medical, administrative or pharmaceutical reasons by principal investigator or sponsor.

### Safety

All serious adverse events (SAE) during the study period, whether or not considered by the Investigator to be related to study treatment, must be reported by fax to the central data management office (ClinAssess GmbH fax +49-2171-3633655) within 24 hours using the completed SAE report and Case Report Form (CRF). The principal investigator is responsible for the management of the safety reporting requirements according to the local regulations and guidelines. Copies of all report submissions by the principal investigator to regulatory authorities (BfArM and accordingly Paul-Ehrlich-Institute, http://www.bfarm.de, http://www.pei.de) and to the ethical committee that has approved the study will be provided to the pharmacovigilance department of the license holders of the study drugs. If necessary, additional information and clarifications on cases will be forwarded to the license holders by the principal investigator.

### Data quality assurance

All patient data are collected in the central database of the data centre at ClinAssess GmbH and the patient identifiers are kept confidential. Computerized and visual consistency checks will be performed on newly entered forms; queries will be issued in case of inconsistencies.

### Monitoring and source data verification

The sponsor will perform on-site monitoring with clinical research associates. The monitoring visits frequency will be adapted according to the site accrual. The aim of on-site visits will be:

• Adherence to recruitment rate

• Adherence to eligibility criteria

• To evaluate the local facilities available to the responsible investigator for performing clinical trials and to comply to all requirements of the present protocol

• Adherence to scheduled examination and evaluation appointments

• Existence of written informed consent

• Integrity of study documentation

• To assess the consistency of the data reported on the CRF with the source data (source data verification)

### Statistical analysis

Statistical analyses will be performed according to the intention-to-treat principle, i.e. all eligible patients will be included in the analysis in the arm to which they were randomized independently of whether they received the assigned treatment or not. A second analysis (according-to-protocol) will include all patients receiving at least 3 cycles of first-line chemotherapy. Toxicity analysis will be conducted in all patients receiving at least one application of study medication.

The primary objective of the study is to examine two hierarchically ordered hypotheses on the comparative efficacy and toxicity. The primary efficacy endpoint is the time to failure of strategy (TFS). The objective is to show non-inferiority of the experimental arm. The primary toxicity analysis is performed confirmatively if (and only if) non-inferiority with respect to TFS was shown. This hierarchically ordered approach precludes an inflation of the overall type I error in case of more than one study hypothesis. The primary safety endpoint is a toxicity score defined as the mean number of NCI CTC grade 2 - 4 findings per cycle during the whole TFS period. Only symptomatic toxicities will be taken into account, and not any events that are manifested as laboratory abnormality or change only. With respect to toxicity the objective is to show superiority of the experimental arm.

The primary endpoints will be analyzed confirmatively with a global level of significance p ≤ 0.05 (one-sided). All other parameters will be estimated in a descriptive analysis with report of means, medians, ranges and confidence intervals. All additional p-values will be estimated exploratorily without adjustment of the level of significance, using two-sided test procedures.

Demographic and prognostic baseline measures will be analyzed for heterogeneity between the two treatment arms. Clinical and laboratory toxicity graded according NCI CTC (version 4.0) will be collected for all patients. Quality of life will be measured using the EORTC QLC-C30 questionnaire. Categorical data comparisons between treatment arms will be performed applying Fisher's exact test, chi-square test and Mantel-Haenszel test, as appropriate. Event-related data (TFS, PFS, OS) will be reported according to the life-table method (Kaplan and Meier) and compared using the logrank test. In case of non-conformity with proportional hazard assumptions the generalized Wilcoxon signed-rank test may be used as modified by Peto et al. and Prentice et al. [14-16].

Univariate estimation for prognostic factors will be performed as described above. In case of need for multivariate analysis appropriate regression models e.g. logistic regression model, Cox proportional hazard model will be adopted.

In case of ethical need or slow recruitment an interim analysis will be performed, applying a prospective group-sequential design using the alpha error spending function published by Lan and Demets [17,18], implementing an O'Brien and Fleming boundary shape. Final data collection date for primary outcome measure will be December 2013 and the estimated study completion date will be December 2016.

### Sample size

Based on published data on FOLFIRI or CAPIRI plus bevacizumab and with regard to a marginally decreased prognosis due to exclusion of resectable patients, a median time to failure of strategy (TFS) of 10 months will be expected. Assuming equal TFS in both arms and in order to statistically exclude an inferior TFS corresponding to a median of 8 months in the experimental arm, 253 observed failures per group are required, i.e. a total of 506 events, to achieve a power of 80% with a one-sided type I level of 0.05 Under assumption of a recruitment period of 3 years and of a minimum follow-up period of 3 years the number of patients to recruit is 258 per treatment arm and a total of 516 in the study.

### Baseline assessment

Baseline investigation will be performed within 14 days before first application of study medication and include the following items:

• Patient information sheet and consent form informed consent

• Patient history (including tumour parameters, prior treatments, concomitant diseases/treatment and prescribed drugs)

• Physical examination

• Body weight and height

• Vital signs (blood pressure and heart rate)

• ECOG-Performance status

• Quality of life questionnaire (EORTC QLQ-C30)

• Haematology and differential blood count

• Serum chemistry (including sodium, potassium, calcium, creatinine, urea, uric acid, estimated creatinine clearance, lactate-dehydrogenase (LDH), c-reactive protein (CRP), bilirubin, alkaline phosphatase (AP), aspartate aminotransferase (AST), alanine aminotransferase (ALT), Gamma-glutamyl transpeptidase (GGT), total protein, albumin, international normalized ratio (INR), activated partial thromboplastine time (APTT)

• Tumour Markers (Carcionembryonic antigen (CEA), carbohydrate antigen 19-9 (CA 19-9)

• Urine analysis (dipstick)

• Pregnancy test in childbearing potential women

• ECG

• Cardiac ultrasound

• CT scan of thorax and abdomen within 4 weeks before randomization (definition of target lesions according to RECIST criteria version 1.1)

• Bone scan and/or x-ray if clinically indicated

### Assessments during study treatment phase

#### At day 1 every cycle

• Patient history (including symptoms, toxicity, concomitant medication)

• physical examination

• Body weight

• Vital signs (blood pressure and heart rate)

• ECOG-Performance status

• Haematology and differential blood count

• Serum chemistry (including sodium, potassium, calcium, creatinine, urea, uric acid, estimated creatinine clearance, lactate-dehydrogenase (LDH), c-reactive protein (CRP), bilirubin, alkaline phosphatase (AP), aspartate aminotransferase (AST), alanine aminotransferase (ALT), Gamma-glutamyl transpeptidase (GGT), total protein, albumin, international normalized ratio (INR), activated partial thromboplastine time (APTT)

• Tumour markers such as carcinoembryonic antigen (CEA) and carbohydrate antigen 19-9 (CA 19-9)

• Urine analysis (dipstick)

#### After 3 cycles

• CT scan of thorax and abdomen (evaluation of target lesions according to RECIST criteria version 1.1)

• Serum chemistry (including sodium, potassium, calcium, creatinine, urea, uric acid, estimated creatinine clearance, lactate-dehydrogenase (LDH), creactive protein (CRP), bilirubin, alkaline phosphatase (AP), aspartate aminotransferase (AST), alanine aminotransferase (ALT), Gamma-glutamyl transpeptidase (GGT), total protein, albumin, international normalized ratio (INR), activated partial thromboplastine time (APTT)

• Tumour markers CEA and CA 19-9

• Quality of life questionnaire (EORTC QLQ-C30)

### End of study assessment or discontinuation of treatment

• Patient history (including symptoms, toxicity, concomitant medication)

• Physical examination

• Body weight

• Vital signs (blood pressure and heart rate)

• ECOG-Performance status

• Quality of life questionnaire (EORTC QLQ-C30)

• Haematology and differential blood count

• Serum chemistry (including sodium, potassium, calcium, creatinine, urea, uric acid, estimated creatinine clearance, lactate-dehydrogenase (LDH), creactive protein (CRP), bilirubin, alkaline phosphatase (AP), aspartate aminotransferase (AST), alanine aminotransferase (ALT), Gamma-glutamyl transpeptidase (GGT), total protein, albumin, international normalized ratio (INR), activated partial thromboplastine time (APTT)

• Tumour markers CEA and CA 19-9

• Urine analysis (dipstick)

• CT scan of thorax and abdomen (evaluation of target lesions according to RECIST criteria version 1.1)

• ECG

### Assessment during follow up period

• Survival information

• Delayed toxicity (first follow-up only)

### Translational research project

Tumour tissue samples from primary tumours and/or metastatic sites are anonymized, collected and stored in a central tumour bank at the Department of Medical Oncology and Department of Pathology, University of Munich. A systematic translational research project with analysis of immunohistochemical, protein- and gene-expression with regard to VEGFR- and EGFR-pathway will be performed in available specimen.

Peripheral blood samples (10 ml of EDTA anticoagulated peripheral blood) for gene-polymorphism analysis with special regard to VEGFR- and EGFR-pathway are taken additionally. Blood samples are stored centrally in the tumour bank at the Department of Medical Oncology, University of Munich.

## Study medication

### Capecitabine

Capecitabine is an oral fluoropyrimidine carbamate rationally designed to generate 5-Fluorouracil preferentially in tumour tissue through exploitation of high intratumoural concentrations of thymidine phosphorylase (TP). TP is found in significantly increased concentrations in a wide range of tumour types, including colorectal, breast and gastric cancers, compared to normal tissue [19]. Previous human pharmacokinetic studies have shown almost complete absorption through the gastro-intestinal wall after oral administration. Direct intestinal exposure to 5-FU is thereby avoided. Capecitabine is metabolized to 5-FU via a three-step enzymatic cascade, with the final conversion to 5-FU mediated by TP [20].

### Irinotecan

Irinotecan is a semisynthetic analogue of the natural alkaloid camptothecin and its active metabolite SN-38 inhibits the enzyme topoisomerase-1. This leads to inhibition of both DNA replication and transcription. SN-38 is inactivated by the enzyme UGT1A1 by glucuronidation, so that patients with UGT1A1 polymorphism are likely to develop severe irinotecan-related toxicities such as diarrhea and neutropenia [21].

### Bevacizumab

Bevacizumab is a humanized monoclonal antibody targeting the vascular endothelial growth factor (VEGF or VEGF-A) playing a central role in signalling pathways controlling tumour blood vessel development and survival. Interruption of this pathway prevents the formation of new blood vessels and normalizes existing tumour blood vessels and vessel permeability allowing cytotoxic drug access into the tumour [22,23].

### Treatment program

The experimental arm (arm A) of the trial consists of the following regimen: capecitabine (1250 mg/m2 BID day 1-14) plus bevacizumab 7.5 mg/kg over 90 minutes on day 1 every three weeks. Treatment continuation is intended until disease progression or development of toxicity. In case of progression escalation to standard chemotherapy with CAPIRI + bevacizumab is provided.

The control arm (arm B) of the trial consists of capecitabine (800 mg/m2 BID day 1-14), irinotecan 200 mg/m2 on day 1 and bevacizumab 7.5 mg/kg over 90 minutes on day 1 every three weeks (Arm B). Treatment continuation is intended until disease progression or development of toxicity. After 6 months of treatment or case of irinotecan-associated toxicity, de-escalation to capecitabine plus bevacizumab is recommended. In case of disease progression after treatment de-escalation, re-escalation to the combination chemotherapy is possible.

## Discussion

Presently, three clinically distinct groups of metastatic colorectal cancer patients can be differentiated: 1) patients with resectable disease; 2a) patients who require conversion chemotherapy or (2b) patients with highly symptomatic or rapidly progressive disease; 3) patients with disseminated metastasis and mostly asymptomatic disease. While only few studies have engaged to investigate these subgroups separately, most clinical trials were performed in the whole population of mCRC patients.

It appears that group 3 represents the largest subgroup. For this reason, several clinical studies have produced results acceptable for this subgroup. Patients in this group do not clearly benefit from rapid remission induction and may rather profit from prolonged disease control.

A previous report by Grothey et al. analysed two trials where patients either received chemotherapy alone (N9741-trial: FOLFOX vs. IFL) or chemotherapy plus bevacizumab (AVF2107g: IFL + bevacizumab versus Bevacizumab alone) [3]. In both trials, the superior regimen improved PFS and OS regardless of tumour response. This led to the conclusion that tumour response in mCRC is not a necessary determinant of therapeutic benefit. This conclusion can be extended to the point that most probably a large fraction of patients simply benefits from non-progressive disease. If disease control becomes an important endpoint, then trials have to be designed where strategies including reduced treatment intensity may reach this goal in a defined population of group-3 patients.

The present trial investigates an escalation strategy where patients start with a well tolerated single-agent chemotherapy (capecitabine) combined with bevacizumab. Patients treated on this study arm are allowed to escalate to CAPIRI plus bevacizumab once disease progression occurs. This escalation strategy is compared to a control arm where patients receive first-line chemotherapy with CAPIRI plus bevacizumab. After 6 months of treatment or if intolerable toxicity is faced, treatment can be de-escalated to capecitabine plus bevacizumab. In case of progression, it can later be re-escalated to CAPIRI plus bevacizumab. This innovative approach therefore offers two strategies which allow a flexible response to the individual requirements of the patient. The study seeks to demonstrate that patients attributed to group 3 have an adequate treatment benefit with a well tolerated low-intensity first-line regimen provided that further treatment is appropriately applied if necessary. As described in the above the CAPIRI regimen with a dose of capecitabine 800 mg/m^2^, irinotecan 200 mg/m^2 ^previously investigated in two AIO randomized phase II trials is expected to have an acceptable toxicity profile [7,11].

In the pre-antibody era, an escalation strategy has already been investigated in the CAIRO (CApecitabine, IRinotecan, and Oxaliplatin in advanced colorectal cancer) study. Koopmann et al. reported a trial arm where treatment was escalated in sequential steps from capecitabine (1^st^-line) to irinotecan (2^nd^-line) to CAPOX (3^rd^-line) [9]. Comparison with the sequential application of CAPIRI (1^st^-line) and CAPOX (2^nd^-line) led to the interpretation that "combination treatment does not significantly improve overall survival compared with the sequential use of cytotoxic drugs" [9].

Another study of the pre-antibody era was the MRC-FOCUS trial. This study compared arm A) 5-FU/FA followed by irinotecan at progression to arm B) 5-FU/FA followed by combination chemotherapy and to arm C) combination chemotherapy from the onset. Also this large study challenged the assumption that in a non-curative setting maximum tolerable treatment must necessarily be used first-line [24].

Comparable results were reported by Bouché et al. who compared the sequence LV5FU2 followed by FOLFOX (2^nd^-line) followed by FOLFIRI (3^rd^-line) to the more conventional sequence of FOLFOX followed by FOLFIRI (2^nd^-line) [25]. Again, this trial confirmed the notion that initially intensive regimens do not induce a superior outcome compared to well tolerated single-agent first-line strategies. However, it is important to state that this conclusion is true most likely for patients treated within group 3.

Since the CAIRO- and the MRC-FOCUS trial, perspectives on curability of disease have changed specifically with regard to the interpretation of surgical respectability [26]. Also the introduction of antivascular and anti-EGFR treatment strategies had a marked impact on treatment outcome. The present trial picks up the theme of escalation chemotherapy, investigates this strategy specifically in group-3 patients and analyses the effect of chemotherapy in the setting of concomitant antiangiogenic therapy with bevacizumab.

In the control arm of our trial, patients may de-escalate treatment from initial combination therapy with CAPIRI plus bevacizumab to a maintenance therapy with capecitabine plus bevacizumab. De-escalation is planned after 6 months of treatment. However it can take place earlier in case of treatment-associated toxicity. A 6-month duration of first-line combination chemotherapy has been described in several other trials [27]. Clearly, the optimal time point of de-escalation and the optimal maintenance regimen still have to be defined. The present trial will contribute to this important topic. Re-escalation from single-agent maintenance to combination chemotherapy is another important aspect of this strategy. Results previously obtained from the OPTIMOX1 trial indicate that reintroduction of chemotherapy had an independent and significant impact on overall survival [28].

The present trial does not simply investigate two treatment regimens, but rather compares two strategies of treatment [29]. For this reason, time to failure of strategy (TFS) was chosen as a primary endpoint of the study. TFS is defined as the time between randomization and final failure of CAPIRI-Bev treatment in both treatment arms. This end point allows a wide range of options to manage mCRC patients while remaining on study thus providing a more complete assessment of a strategy's benefit [29].

In case of comparable TFS times, toxicity will be evaluated according to NCI CTC in a predefined score system using symptomatic grade 2-4 toxicities per cycle. This innovative design allows to analyze both, the therapeutic strategies and the associated toxicity.

When this study is performed, it is necessary to also define those patients who are clearly not candidates for this trial. Due to the selected control arm, all patients considered for the trial must be able to tolerate first-line combination chemotherapy with CAPIRI. The present study has not been specifically designed for elderly or frail patients and clearly excludes those with an ECOG performance status ≥ 2. It also excludes patients who present with symptomatic tumour disease. These patients need a fast treatment response which can best be achieved by intensive combination therapy.

While the present trial requires that parameters of the EGFR-pathway are determined, KRAS mutation of the tumour does not affect treatment within the trial. Since treatment efficacy of bevacizumab so far is not considered to depend on KRAS mutational status and since patients receive bevacizumab in both treatment arms, also patients with KRAS mutation can take part in the study.

## Conclusion

The AIO KRK 0110 trial is designed for patients with disseminated, but asymptomatic mCRC who are not potential candidates for surgical resection of metastasis. Two bevacizumab-based strategies are compared: one starting as single-agent chemotherapy (Cape-Bev) allowing escalation to CAPIRI-Bev and another starting with combination chemotherapy (CAPIRI-Bev) and allowing de-escalation to Cape-Bev and subsequent re-escalation if necessary.

## List of abbreviations

AE: Adverse event; ALT: Alanin-Aminotransferase; AST: Aspartat-Aminotransferase; AP: Alkaline phosphatise; APTT: Activated patial thromboplastin time; Bev: Bevacizumab; BfArM: Bundesinstitut für Arzneimittel und Medizinprodukte; CA 19-9: Carbohydrate Antigen 19-9; CAPOX: Capecitabin und Oxaliplatin; CEA: Carcinoembryonic antigen; CR: Complete Remission; CRF: Case Report Form; CRP: C-reactive protein; CT: Computertomography; DPD: Dihydropyrimidin-Dehydrogenase; ECG: Electrocardiogram; ECOG: Eastern Cooperative Oncology Group; EGFR: Epidermal Growth Factor Receptor; EORTC European organisation for research and treatment of cancer, FOLFIRI, Folinc acid, 5-Fluorouracil and irinotecan; FOLFOX, 5-Fluorouracil, leucovorine und oxaliplatin; FU: 5-Fluorouracil; FUFOX: Fluorouracil, folinic acid and oxaliplatin; GCP: Good clinical practice; ICH: International Conference on Harmonization; LDH: Lactate-dehydrogenase; LVEF: Left ventricle ejection fraction; mCRC: Metastatic colorectal cancer; NCI-CTCAE: National Cancer Institute Common Terminology Criteria for Adverse Events; NSAIDS: Nonsteroidal Antiinflammatory Drugs; NYHA: New York Heart Association; ORR: Overall response rate; OS: Overall survival; PD: Progressive disease; PFS: Progression-free survival; PR: Partial remission; RECIST: Response Evaluation Criteria in Solid Tumours; SD: Stable disease; SNP: Single Nucleotide Polymorphism; SAE: Severe adverse event; SUSAR: Suspected unexpected adverse reaction; TFS: Time to failure of strategy; TTP: Time to progression; QLQ: Quality of life questionnaire; ULN: Upper Limit of Normal; VEGF: Vascular Endothelial Growth Factor; VEGFR: Vascular Endothelial Growth Factor Receptor; vs., versus; XELIRI: Xeloda (capecitabin) and irinotecan.

## Competing interests

VH: Honoria for talks and advisory boards (Roche), research funding for clinical trial (Roche). All other authors declare that they have no competing interests.

## Authors' contributions

CG and VH drafted the manuscript. VH, SS and CG wrote the original protocol for the study. AH performed the statistical analysis for the study design and participated in drafting the manuscript. SS, FK, UVK, JM, MB, CD, MS, CT, JFR, CS, DPM, NM and PA are providing study material or patients and participated in drafting the manuscript. All authors read and approved the final manuscript.

## Pre-publication history

The pre-publication history for this paper can be accessed here:

http://www.biomedcentral.com/1471-2407/11/367/prepub
